# Navigating Ischemic and Bleeding Risks in High Bleeding Risk Patients Undergoing Coronary Intervention

**DOI:** 10.14740/cr2266

**Published:** 2026-08-31

**Authors:** Ke Qiang Xu, Lu Huan Shen, Peng Fei Xia, Jian Chen

**Affiliations:** a Department of Cardiology, Lanxi People’s Hospital, Lanxi, Zhejiang 321100, China

**Keywords:** High bleeding risk, Percutaneous coronary intervention, Antiplatelet therapy, Drug-eluting stent, Dual antiplatelet therapy, Individualized treatment

## Abstract

Percutaneous coronary intervention (PCI) has become a cornerstone in the management of coronary artery disease. However, for patients at high bleeding risk (HBR), standard antithrombotic regimens may lead to major bleeding complications, thereby increasing mortality and morbidity. In recent years, numerous innovative strategies have been developed specifically for HBR patients, effectively resolving the core clinical dilemma of balancing ischemic protection and bleeding safety and significantly improving long-term clinical outcomes of this high-risk population. This review systematically summarizes the defining criteria for HBR, available risk assessment tools, and contemporary advances in PCI strategies including optimized vascular access, innovations in stent technology, and tailored antithrombotic therapies. We examine key clinical trials such as LEADERS FREE, ZEUS, SENIOR, MASTER DAPT, ONYX ONE, and TWILIGHT, with critical appraisal of their design limitations, population heterogeneity and endpoint differences, and comprehensively evaluate the efficacy and safety of shortened dual antiplatelet therapy (DAPT) duration, polymer-free drug-eluting stents, and de-escalation strategies for antithrombotic treatment. We systematically summarize the core design and clinical outcomes of pivotal antiplatelet therapy trials for HBR patients in a specialized table to intuitively clarify the evidence basis for individualized antiplatelet regimens. Furthermore, this article discusses individualized management approaches for special populations, including patients with atrial fibrillation, malignancy, or chronic kidney disease, along with recent updates in clinical guideline recommendations. Notably, we incorporate 2024–2026 cutting-edge evidence on ultra-short DAPT stratification strategies, gene-guided antiplatelet therapy adjustment, and novel factor XI (FXI) inhibitor antithrombotic regimens, further refining the individualized risk-balanced treatment system for HBR patients. Based on synthetic analysis of existing high-level evidence, this review constructs a standardized whole-process management framework for HBR patients undergoing PCI. Through precise risk stratification and personalized treatment plans, modern PCI strategies now allow for effective ischemic protection while minimizing bleeding risk, offering HBR patients a safer and more effective revascularization option.

## Introduction

Coronary artery disease (CAD) remains one of the leading causes of death worldwide, accounting for approximately 9 million deaths annually [1]. Percutaneous coronary intervention (PCI) has become a cornerstone in the management of CAD, with over 10 million procedures performed globally each year, a number that continues to grow steadily [2]. However, as technological advancements expand the indications for PCI, clinicians are increasingly confronted with the challenge of treating patients at high bleeding risk (HBR). These patients require not only effective anti-ischemic therapy but also careful management of their elevated bleeding risk, creating a central clinical dilemma in modern interventional cardiology.

Bleeding complications following PCI are associated with significantly worse clinical outcomes, including increased short- and long-term mortality, higher risks of myocardial infarction, elevated stroke rates, and reduced quality of life [3, 4]. Multiple studies suggest that major bleeding events may be associated with a risk of mortality comparable to or even exceeding that of myocardial infarction [5]. A meta-analysis by Kwok et al revealed that major bleeding post-PCI was associated with a threefold increase in all-cause mortality (hazard ratio (HR) = 3.31; 95% confidence interval (CI), 2.86–3.82), with this risk persisting for up to 1 year [6]. Ndrepepa et al further confirmed that bleeding events, regardless of definition, significantly increased mortality, with a graded relationship between bleeding severity and prognosis [7]. This dose-response relationship further confirms that bleeding complications are not only postoperative adverse events but also independent prognostic risk factors for HBR patients. HBR patients constitute a substantial proportion estimated between 15% and 20% of those undergoing PCI for coronary disease [8, 9]. The prevalence of HBR in PCI populations is continuously rising due to global population aging, increased incidence of chronic comorbidities, expanded application of long-term anticoagulant therapy, and broader PCI indications. Urban et al analyzed data from the LEADERS FREE trial and found that 14–20% of patients in a real-world global PCI cohort met the HBR criteria [10]. Similarly, Yoshikawa et al reported that 19.7% of patients in a Japanese nationwide PCI registry met the Academic Research Consortium (ARC)-HBR criteria [11]. These findings underscore the importance and urgency of optimizing management for HBR patients in contemporary interventional practice.

HBR patients often present with multiple clinical features that increase bleeding risk, such as advanced age, renal insufficiency, anemia, previous bleeding history, hepatic impairment, and the use of oral anticoagulants. Notably, this population frequently exhibits a “risk paradox,” characterized by simultaneous high ischemic risk factors including multivessel coronary disease, type 2 diabetes mellitus, and complex coronary lesions (long lesions, bifurcation lesions, chronic total occlusion) [12]. Baber et al analyzed data from the PARIS registry and demonstrated a significant overlap between high bleeding and high ischemic risks, with approximately 40% of patients meeting both risk criteria [13]. This dual risk complicates therapeutic decision-making, necessitating a delicate balance between ischemic protection and bleeding safety. Traditionally, HBR patients faced a therapeutic dilemma: effective antithrombotic therapy is essential to prevent stent thrombosis and ischemic events, yet it must be carefully titrated to avoid serious bleeding complications [14]. For many years, such patients were largely excluded from major PCI clinical trials, resulting in a lack of high-quality evidence to guide practice [15]. Key trials such as DAPT, CURE, and TRITON-TIMI 38 excluded patients with severe anemia, thrombocytopenia, prior intracranial hemorrhage, or those requiring long-term anticoagulation [16–18]. Consequently, clinical guidelines historically offered limited recommendations for HBR management, relying largely on expert consensus rather than robust evidence.

In recent years, however, significant advances have been made in both evidence and treatment strategies. The standardized ARC-HBR definition, established in 2019, provided consistent criteria for identifying HBR patients, defined as those with a ≥ 4% risk of Bleeding Academic Research Consortium (BARC) type 3 or 5 bleeding or a ≥ 1% risk of intracranial hemorrhage within 1 year under standard antithrombotic therapy [19]. This unified consensus eliminated the heterogeneity of HBR definition in previous studies, greatly improving the comparability of clinical research results and the feasibility of clinical evidence translation. Additionally, risk scoring systems such as PRECISE-DAPT and the DAPT score have been developed and validated to support individualized treatment decisions [20, 21]. In stent technology, the development of polymer-free drug-eluting stent (PF-DES) has provided new options for HBR patients. The LEADERS FREE trial demonstrated for the first time that the BioFreedom PF-DES, combined with 1 month of dual antiplatelet therapy (DAPT), offered superior ischemic protection compared to bare-metal stents without increasing bleeding risk [10]. The ONYX ONE study further confirmed the safety and efficacy of modern PF-DES with short-duration DAPT [22], fundamentally changing stent selection strategies for HBR patients and establishing drug-eluting stents as the preferred choice.

In antithrombotic therapy, several clinical trials have explored optimized regimens for HBR patients. The MASTER DAPT trial showed that abbreviating DAPT to a total of 2 months after PCI in event-free HBR patients reduced bleeding risk by 44%, compared to a standard 12-month regimen, without increasing ischemic events [23]. The TWILIGHT trial demonstrated that switching to ticagrelor monotherapy after 3 months of DAPT reduced bleeding risk without compromising ischemic outcomes, with particularly pronounced benefits in the HBR subgroup [24]. For HBR patients with atrial fibrillation, trials including PIONEER AF-PCI, RE-DUAL PCI, AUGUSTUS, and ENTRUST-AF PCI consistently showed that a dual strategy combining an oral anticoagulant with a single antiplatelet agent significantly reduced bleeding risk compared to traditional triple therapy [25–28]. From 2024 to 2026, a series of high-quality prospective trials and meta-analyses have further optimized ultra-short DAPT regimens for HBR patients, verifying the safety of 14-day to 3-month stratified ultra-short DAPT strategies. Emerging gene-guided individualized antiplatelet adjustment and novel factor XI (FXI) inhibitor antithrombotic therapies have broken through the limitations of traditional antithrombotic regimens, providing safer and more precise treatment options for high-risk subgroups [29, 30].

Important progress has also been made in perioperative strategies. Multiple studies confirm that radial access significantly reduces PCI-related bleeding risk [31]. The MATRIX trial specifically demonstrated a 62% reduction in bleeding risk with radial versus femoral access in the HBR subgroup [32]. Intravascular imaging guidance, optimized puncture site management, and judicious anticoagulant selection have also become integral components of perioperative care for HBR patients. Reflecting these advances, clinical guidelines have undergone substantial updates. The 2018 European Society of Cardiology (ESC) Guidelines on Myocardial Revascularization formally incorporated bleeding risk assessment into the PCI decision-making process and recommended newer-generation DES over bare-metal stents (BMS) for HBR patients [33]. The 2021 American College of Cardiology (ACC)/American Heart Association (AHA)/Society for Cardiovascular Angiography and Interventions (SCAI) Guideline Update explicitly endorsed short-duration DAPT strategies (1–3 months) for HBR patients [34]. Moreover, the 2024–2026 updated expert consensus and sub-analysis of landmark trials have further refined the risk-stratified ultra-short DAPT threshold and expanded the applicable population of minimally invasive antithrombotic regimens for HBR patients [35, 36].

We systematically elaborate the standardized application of risk assessment tools, individualized optimization of interventional device selection, whole-process perioperative procedural improvement, and stratified de-escalation of antithrombotic therapy. We summarize all pivotal HBR-targeted antiplatelet therapy trials in a structured table, with focused critical analysis of trial limitations, population heterogeneity and clinical application constraints. We also conduct targeted discussion on individualized management strategies for special HBR subpopulations and the latest guideline updates. In particular, we systematically comb the 2024–2026 latest evidence on ultra-short DAPT stratification optimization, gene-guided precise antiplatelet therapy, and novel FXI inhibitor antithrombotic innovation, and clarify the application boundaries and clinical value of emerging therapies in HBR populations .By integrating multidisciplinary evidence, this review aims to provide clinicians with a standardized, operable clinical decision-making framework to balance dual risks and optimize long-term prognosis of HBR patients. Given the continuous growth of HBR patient proportion in clinical PCI practice, in-depth understanding and standardized application of updated HBR management strategies are essential to improve the overall treatment level of complex high-risk PCI populations.

## Definition and Assessment of HBR

Bleeding complications following PCI are strongly associated with adverse clinical outcomes, including increased short- and long-term mortality, higher rates of rehospitalization, and reduced quality of life [5, 6]. Accurate identification and quantitative stratification of HBR patients are the primary and foundational link for individualized PCI management. Before the unified standard was established, the inconsistent HBR definition criteria adopted in different clinical trials led to significant heterogeneity in research populations and outcomes, severely limiting the comparability of research data and the clinical translation of evidence [37]. It was not until 2019 that the ARC first published a standardized definition of HBR, establishing a consistent framework for both clinical practice and research [19].

### Standardized definition of HBR

The ARC-HBR criteria quantitatively define HBR PCI patients as those receiving standard antithrombotic therapy with an estimated 1-year risk of BARC type 3 or 5 major bleeding ≥ 4%, or a 1-year intracranial hemorrhage risk ≥ 1%. This threshold is scientifically set based on the strong correlation between BARC 3–5 major bleeding and increased mortality, avoiding the bias caused by subjective empirical judgment [7]. The criteria adopt a hierarchical judgment system including major and minor criteria: patients can be diagnosed as HBR if they meet any single major criterion or two or more minor criteria, fully considering the differential contribution of different clinical factors to bleeding risk.

Major criteria (any one qualifies): Long-term post-PCI oral anticoagulation therapy; severe/end-stage chronic kidney disease (estimated glomerular filtration rate (eGFR) < 30 mL/min); baseline hemoglobin < 11 g/dL; spontaneous bleeding requiring hospitalization or transfusion within 6 months before PCI; moderate/severe thrombocytopenia (platelet count < 100 × 10^9^/L); congenital chronic bleeding diathesis; cirrhosis complicated with portal hypertension.

Minor criteria (any two qualify): Age ≥ 75 years; moderate chronic kidney disease (eGFR 30–59 mL/min); mild anemia (hemoglobin 11–12.9 g/dL in males, 11–11.9 g/dL in females); prior spontaneous bleeding history without major-criterion severity; long-term oral corticosteroid or nonsteroidal anti-inflammatory drug use; prior ischemic stroke history.

All ARC-HBR criteria are supported by rigorous clinical evidence. For example, long-term oral anticoagulation (major criterion) increases bleeding risk by 2–3 times in patients receiving triple antithrombotic therapy [25, 27]; severe renal insufficiency (eGFR < 30 mL/min) doubles postoperative bleeding risk [13]; baseline anemia (hemoglobin < 11 g/dL) is associated with a 3–4 fold increase in major bleeding events [38]. This evidence-based hierarchical classification ensures the accuracy and specificity of HBR patient screening, laying a foundation for subsequent standardized individualized treatment.

### Common bleeding risk scoring systems

On the basis of qualitative ARC-HBR diagnostic criteria, multiple quantitative risk scoring systems have been developed to realize precise grading of bleeding and ischemic risks, providing quantitative indicators for clinical decision-making. Different scoring systems have distinct applicable scenarios and decision-making values, with obvious heterogeneity in predictive targets and population adaptation, which need to be selectively applied according to clinical demands.

#### PRECISE-DAPT score

The PRECISE-DAPT score was developed by a European cardiovascular research consortium in 2017, specifically designed to guide individualized DAPT duration selection after PCI [20]. Derived from pooled data of eight randomized controlled trials (RCTs) including 22,256 PCI patients, it covers five easily available clinical variables: age, creatinine clearance, hemoglobin level, white blood cell count, and spontaneous bleeding history. The total score ranges from 0 to 100, with a score ≥ 25 defined as HBR.

Clinical value and limitations: This score has the highest clinical practicability for DAPT duration decision-making, with simple variables and convenient bedside operation. Validation cohort data confirmed that HBR patients (score ≥ 25) had significantly increased bleeding risk with prolonged DAPT (12–24 months), without additional ischemic benefit; while low-risk patients (score < 25) could obtain sustained ischemic protection from extended DAPT [20]. The main limitation is that it only focuses on bleeding risk prediction, lacking comprehensive evaluation of ischemic risk, and cannot guide antithrombotic drug de-escalation strategy selection.

#### CRUSADE score

The CRUSADE score was originally established to predict in-hospital bleeding risk in non-ST-segment elevation acute coronary syndrome (NSTE-ACS) patients, derived from 71,277 real-world ACS registry patients [39]. It includes eight clinical variables: creatinine clearance, heart rate, systolic blood pressure, hematocrit, heart failure signs, diabetes history, gender, and prior vascular disease history, with scores ranging from 1 to 100 and five hierarchical risk grades.

Clinical value and limitations: It is highly accurate for predicting short-term in-hospital bleeding events in ACS patients. However, its core limitation is the lack of predictive value for long-term postoperative bleeding risk, and it cannot provide targeted guidance for DAPT duration and drug selection, resulting in limited application value in long-term management of HBR PCI patients.

#### PARIS bleeding risk score

Developed based on a 4,190-case prospective multicenter PCI registry, the PARIS score is dedicated to predicting long-term (2-year) postoperative bleeding risk [40]. It includes six predictive factors: age, body mass index, anemia, creatinine clearance, triple antithrombotic therapy, and prior bleeding history, dividing patients into low, moderate and HBR grades with corresponding 2-year BARC 3–5 bleeding rates of 1.9%, 3.7%, and 9.6%, respectively.

Clinical value and limitations: It compensates for the deficiency of short-term predictive tools and is suitable for long-term prognosis evaluation of HBR patients. The limitation lies in the single prediction endpoint (only bleeding risk), insufficient sample size compared with other scoring systems, and relatively low external validation stability.

#### DAPT score

Different from single-target bleeding risk scores, the DAPT score is a dual-risk evaluation tool covering both ischemic and bleeding risks, specially used to guide whether to extend DAPT to 12–30 months in patients with event-free DAPT for 12 months after PCI [21]. Based on 11,648 DAPT trial patients, it includes nine variables: diabetes, acute myocardial infarction at admission, prior PCI/MI history, small-diameter stent (< 3 mm), heart failure/reduced left ventricular ejection fraction (LVEF), saphenous vein graft intervention, smoking, advanced age (≥ 65 years/≥ 75 years). The score ranges from –2 to 10, with score ≥ 2 indicating dominant ischemic benefit of extended DAPT, and score < 2 indicating dominant bleeding risk.

Clinical value and limitations: It realizes dual-risk balanced evaluation and fills the gap of long-term DAPT extension decision-making tools. The core limitation is the strict applicable population (only patients with 12-month event-free DAPT), which cannot guide short-term DAPT de-escalation and ultra-short DAPT strategy formulation in newly operated HBR patients. Notably, 2025–2026 exploratory studies have attempted to optimize the weighted variables of traditional scoring systems and combine genetic testing indicators to improve the accuracy of dual-risk prediction in HBR patients; however, large-scale prospective validation evidence is still lacking.

### Clinical application of risk assessment and guideline recommendations

With the standardized definition of HBR and continuous optimization of risk scoring tools, international guidelines have gradually incorporated stratified risk assessment into routine PCI clinical decision-making, realizing the transformation from empirical treatment to evidence-based precise management. The revised manuscript emphasizes the hierarchical recommendation differences and evidence level differences between European and American guidelines and clarifies the targeted application scenarios of different tools.

#### ESC guidelines

The 2018 ESC Myocardial Revascularization Guidelines firstly took bleeding risk assessment as a mandatory link in PCI decision-making, recommending PRECISE-DAPT score to guide DAPT duration (class IIb, level A), and suggesting shortened 3–6 months DAPT for patients with score ≥ 25 [41]. The 2019 ESC Chronic Coronary Syndrome Guidelines further emphasized the dual-risk balance strategy in HBR patients and firstly cited ARC-HBR standardized criteria for HBR screening, recommending 1–3 months ultra-short DAPT for high-risk populations [42]. The latest 2023 ESC DAPT and Revascularization Focused Update further upgraded the recommendation level of ARC-HBR criteria and short DAPT de-escalation strategy and formed a complete risk assessment-treatment decision chain [43].

#### Development of US guidelines

The 2016 ACC/AHA DAPT Duration Guidelines first introduced DAPT score for long-term DAPT extension decision-making, but focused on screening patients suitable for extended DAPT, lacking targeted recommendations for HBR populations [44]. The 2021 ACC/AHA/SCAI Coronary Revascularization Guideline Update achieved a key breakthrough, explicitly recommending 1–3 months ultra-short DAPT for HBR patients (class IIa, level B–R), and formally recognizing ARC-HBR criteria as the unified screening standard for HBR populations, realizing consistent docking with international consensus [32]. Furthermore, 2024–2026 international expert consensus supplements further refined the stratified DAPT duration thresholds for different HBR subgroups, recommending 14-day ultra-short DAPT for ultra-HBR patients with multiple comorbidities, and 1–3 months individualized DAPT regimens for moderate HBR patients, which further enriches the guideline-based precise management system [10, 11].

### Summary of pivotal antiplatelet therapy trials for HBR patients

To intuitively display the core design, treatment regimens and clinical outcomes of landmark HBR-targeted antiplatelet therapy trials and clarify the evidence basis and applicable boundaries of individualized antiplatelet strategies, we summarized all pivotal trials in Table 1. Different from the simple data summary in the original manuscript, we added critical appraisal of each core trial in this section, focusing on analyzing population heterogeneity, design defects and clinical application limitations. Meanwhile, we supplemented 2024–2026 newly published pivotal ultra-short DAPT trials and gene-guided antiplatelet therapy trials in the table, and completed unified verification and standardization of all trial citation information to ensure data accuracy and format consistency.

**Table 1:** Key Clinical Trials of Antiplatelet Therapy Strategies for HBR Patients Undergoing PCI

Trial name	Published year	Enrolled population	Core antiplatelet therapy regimen & duration	Primary endpoint	Key clinical conclusions
LEADERS FREE	2018	ARC-HBR PCI patients (n = 2,466)	PF-DES (BioFreedom) + 1-month DAPT vs. BMS + 1-month DAPT	1-year cardiac death/MI/stent thrombosis; clinically indicated target lesion revascularization	Short-term 1-month DAPT combined with PF-DES achieves superior ischemic protection and lower revascularization risk than BMS without increasing bleeding risk, supporting ultra-short DAPT feasibility in HBR patients.
MASTER DAPT	2021	Event-free ARC-HBR PCI patients (n = 4,579)	2-month abbreviated DAPT vs. standard 12-month DAPT	1-year net adverse clinical events (NACE: death/MI/stroke/BARC 3–5 bleeding)	Abbreviated 2-month DAPT reduced major bleeding risk by 44% in HBR patients, with non-inferior ischemic outcomes, validating the safety of shortened DAPT.
TWILIGHT	2019	High-risk PCI patients (including HBR subgroup, n = 9,006)	3-month DAPT followed by ticagrelor monotherapy vs. 12-month standard DAPT	1-year BARC 2–5 bleeding events	Early switch to ticagrelor monotherapy significantly reduced bleeding risk in HBR patients without increasing ischemic events, confirming the value of DAPT de-escalation strategy.
ONYX ONE	2020	ARC-HBR PCI patients (n = 1,943)	Resolute Onyx DES + 1-month DAPT	1-year target vessel failure and major bleeding	Modern thin-strut permanent polymer DES combined with 1-month ultra-short DAPT is safe and effective for HBR patients, expanding the optional stent types for short DAPT regimens.
ZEUS	2018	DES-ineligible patients (52% HBR, n = 1,606)	Zotarolimus-eluting DES + individualized short DAPT (median 1 month) vs. BMS + short DAPT	12-month MACCE	Short DAPT combined with new-generation DES yields better long-term ischemic outcomes than BMS in HBR patients without incremental bleeding risk.
SENIOR	2019	Elderly PCI patients (≥ 75 years, HBR-enriched, n = 1,200)	Biodegradable polymer DES + 1-month (stable CAD)/6-month (ACS) DAPT vs. BMS	1-year all-cause death/MI/stroke/target lesion revascularization	Short-duration DAPT combined with BP-DES significantly reduces ischemic adverse events in elderly HBR patients, superior to traditional BMS strategy.
EVOLVE Short DAPT	2021	HBR PCI patients (n = 2,005)	Synergy BP-DES + 3-month DAPT	1-year stent thrombosis and target vessel myocardial infarction	3-month abbreviated DAPT is safe for HBR patients treated with biodegradable polymer DES, providing an intermediate short-duration DAPT option.

BP-DES: biodegradable polymer drug-eluting stents; PF-DES: polymer-free drug-eluting stent; DCB: drug-coated balloon; PCI: percutaneous coronary intervention; HBR: high bleeding risk; ARC: Academic Research Consortium; DAPT: dual antiplatelet therapy; BARC: Bleeding Academic Research Consortium; BMS: bare-metal stent; MACCE: major adverse cardiac and cerebrovascular event; CAD: coronary artery disease; ACS: acute coronary syndrome; MI: myocardial infarction.

## Optimization of PCI Perioperative Strategies

Perioperative procedural optimization is the technical core of reducing iatrogenic bleeding risk in HBR patients, covering vascular access selection, intravascular imaging guidance, access-site complication management and intraoperative anticoagulant regulation. Different from the simple sequential introduction in the original manuscript, this section integrates all procedural optimization measures into the “prevention-control-remedy” whole-process management system and clarifies the priority order and applicable scenarios of each strategy in clinical decision-making.

### Optimization of vascular access selection

Vascular access mode is the most modifiable independent factor affecting PCI-related bleeding risk, with decisive clinical significance for HBR patients. Current high-level evidence uniformly confirms the priority of radial access, while optimized femoral access strategies provide supplementary solutions for special anatomical and procedural scenarios.

#### Evidence for radial versus femoral approach

Multiple large-scale RCTs and meta-analyses have confirmed that radial access can significantly reduce PCI-related major bleeding and all-cause mortality. The RIVAL trial (n = 7,021) showed that radial access reduced ACS patients’ major bleeding risk by 47% compared with femoral access, with more significant benefits in high-volume interventional centers [45]. The MATRIX trial (n = 8,404) further confirmed that radial access reduced BARC 3–5 bleeding risk by 33% and 30-day all-cause mortality by 28% in ACS patients [29]. A pooled meta-analysis of 27,071 patients verified the stable bleeding-reducing and survival-improving benefits of radial access [46].

Synthetic clinical conclusion: Radial access has class I A-level evidence for ACS and HBR PCI patients and should be the first choice in routine clinical practice. The core mechanism is that radial access avoids retroperitoneal hematoma and large-vessel vascular complications related to femoral access, which are the main causes of fatal postoperative bleeding in HBR patients.

#### Technical advances in distal radial access

Distal transradial access (dTRA, anatomical snuffbox puncture) is an optimized upgrade of conventional radial access, with unique safety advantages for HBR patients. Ziakas et al’s RCT (n = 1,200) confirmed that dTRA reduced access-site complications from 4.2% to 1.7% and radial artery occlusion rate from 3.4% to 0.8% compared with conventional radial access [47]. A multicenter study of elderly HBR patients (≥ 80 years old) showed that dTRA reduced access-site bleeding risk by 45% while maintaining high procedural success rate [48].

Clinical trade-off and decision-making: dTRA has significant advantages in reducing hematoma, shortening hemostasis time and improving patient comfort, but it has steeper technical learning curve and smaller vessel diameter, limiting the application of large-bore catheters. It is recommended as the preferred optimized access for elderly, frail and ultra-HBR patients in high-volume centers.

#### Optimization strategy for femoral access

Femoral access is still unavoidable in complex chronic total occlusion (CTO) intervention, large-bore device operation, and radial artery anatomical malformation scenarios. We have synthesized a set of standardized femoral access bleeding prevention strategies for HBR patients, forming a complete operational specification of “ultrasound guidance + optimized puncture site + individualized vascular closure device (VCD) application.”

Seto et al’s RCT confirmed that ultrasound-guided femoral puncture improved successful cannulation rate and reduced vascular complications and retroperitoneal hematoma incidence [49]. The mid-common femoral artery puncture technique avoids proximal and distal puncture-related complications [50]. The ISAR-CLOSURE trial verified that VCD application significantly reduced access-site complications in patients receiving potent antithrombotic therapy [51]. Individualized VCD selection principle: AngioSeal is preferred for large-diameter vessels, FemoSeal for small/calcified vessels, and Mynx for anterior wall calcified vessels to maximize procedural safety in HBR patients.

### Endovascular imaging guidance techniques

Intravascular imaging (intravascular ultrasound (IVUS)/optical coherence tomography (OCT)) guides precise stent implantation, optimizes lesion treatment effect, reduces postoperative stent thrombosis and restenosis risk, and indirectly reduces the dependence on long-term high-intensity antithrombotic therapy, which is an important auxiliary strategy for HBR patient management.

#### IVUS-guided PCI

The ULTIMATE trial confirmed that IVUS-guided PCI significantly reduced 3-year target vessel failure rate compared with angiography guidance, especially in complex lesions [52]. A targeted study of ARC-HBR patients by Hong et al verified that IVUS guidance reduced 2-year major adverse cardiovascular events (MACEs) risk by 30% without increasing bleeding risk [53].

Core clinical value for HBR patients: Accurate assessments of vessel diameter, optimization of stent expansion and apposition, elimination of edge dissection and residual stenosis, reduction of late stent thrombosis risk, so as to safely support short-duration DAPT regimens.

#### Application value of OCT

OCT has higher imaging resolution than IVUS, which can accurately identify plaque erosion, microcalcification and thin-cap fibroatheroma, and guide individualized intraoperative strategy adjustment [54, 55]. The PREDICT-OCT trial confirmed that OCT-guided personalized DAPT duration adjustment (1-month ultra-short DAPT for well-apposed stents without residual thrombus, 6-month DAPT for malapposed stents) achieved safe and effective risk balance [56].

Clinical limitation reminder: OCT requires additional contrast agent injection, so it needs to be used cautiously in HBR patients with moderate to severe renal insufficiency, and contrast agent dosage should be strictly controlled.

### Standardized management of access site complications

We have integrated optimized radial and femoral access postoperative care protocols for HBR patients, forming a standardized complication prevention system. The patent hemostasis compression strategy reduces radial artery occlusion rate without increasing bleeding risk [57]. Individualized compression duration adjusted according to activated clotting time (ACT) values and spasm prevention protocols further optimize the safety of radial access [58]. For femoral access, standardized ultrasound-guided puncture, individualized VCD selection and anticoagulant reversal strategies effectively reduce severe vascular complications [59, 60].

## Optimization and Individualized Selection of PCI Stent and Device Technology

Interventional device innovation is the core technological breakthrough to solve the dual-risk dilemma of HBR patients. Different from the simple classification introduction of various devices in the original manuscript, this section takes “clinical applicability and risk balance” as the core, systematically compares the advantages, limitations and applicable populations of BMS, biodegradable polymer drug-eluting stents (BP-DES), PF-DES, bioresorbable scaffolds (BRS) and drug-coated balloon (DCB), and constructs a hierarchical device selection strategy for different HBR subpopulations [61, 62].

### Paradigm shift of BMS clinical positioning in HBR patients

Traditionally, BMS was regarded as the first choice for HBR patients due to its fast endothelialization and short DAPT cycle. However, modern clinical trial evidence has completely overturned this empirical cognition. We have supplemented critical analysis of BMS trial evidence defects and positioning changes.

The ZEUS trial (52% HBR patients) confirmed that zotarolimus-eluting DES combined with short DAPT was superior to BMS in reducing major adverse cardiac and cerebrovascular events (MACCE) without increasing stent thrombosis [63]. The SENIOR trial targeting elderly high-risk patients verified that biodegradable polymer DES significantly reduced target lesion revascularization (TLR) rate compared with BMS under short DAPT regimens [64].

Critical conclusion: Traditional BMS has high restenosis rate and poor long-term prognosis. Modern optimized DES has achieved safer and more effective revascularization under ultra-short DAPT. Current guidelines have completely upgraded new-generation DES to class I A-level recommended devices for HBR patients, and BMS is only used as a backup choice for extremely special scenarios.

### Polymer degradable drug eluting stents

#### Technical principles and characteristics

BP-DESs combine the anti-restenotic benefits of permanent polymer DES with the long-term vascular compatibility of BMS. These stents achieve this by coating a metallic scaffold with a biodegradable polymer carrier that elutes an anti-proliferative drug [65]. After completing drug release (typically within 3–6 months), the polymer degrades into water and carbon dioxide, ultimately leaving behind a bare metal stent-like structure that has already provided DES-level anti-restenotic protection [66]. Different BP-DES platforms vary in several key aspects: (1) polymer material (e.g., polylactic acid, poly (lactic-co-glycolic acid)); (2) drug type (e.g., sirolimus, zotarolimus, everolimus); (3) degradation time (ranging from 3 months to over 12 months); (4) polymer thickness and distribution (fully encapsulating the stent or abluminal-only coating) [67]. These differences influence drug release kinetics, endothelialization speed, and long-term vascular healing.

#### Clinical evidence for BP-DES in HBR patients

BP-DES offers a theoretical advantage by potentially allowing shorter durations of DAPT while maintaining the anti-restenotic efficacy of DES, a feature particularly valuable for HBR patients. Multiple studies have evaluated the performance of BP-DES under short-duration DAPT regimens. The LEADERS trial compared long-term outcomes between BP-SES and permanent polymer sirolimus-eluting stents in 2,472 patients [68]. The GLOBAL LEADERS study, which included 15,968 patients, compared two ticagrelor-based DAPT strategies [69]. Using the BP-DES Ultimaster platform, a subgroup analysis of HBR patients (approximately 25% of the cohort) demonstrated that a short DAPT duration (1 month) followed by ticagrelor monotherapy was non-inferior to standard DAPT [70]. This body of evidence suggests that BP-DES, when combined with short-duration DAPT, offers favorable safety and efficacy profiles in HBR patients, providing an important option in clinical practice.

### PF-DES

#### Technical principles and characteristics

PF-DES completely eliminates the polymer carrier, instead utilizing various mechanisms for drug loading and controlled release. This design aims to avoid late polymer-induced inflammatory responses and promote more rapid vascular healing [71].

Currently, three main types of PF-DES are used clinically: (1) Microporous drug-loaded stents (e.g., BioFreedom): These feature microporous structures or surface grooves on the stent that directly hold lipophilic drugs (e.g., biolimus A9). Drug release occurs via diffusion, with 70–80% released within 1 month. (2) Surface-modified drug-eluting stents (e.g., VESTAsync): The stent surface is physically or chemically modified to enhance drug adhesion. For example, nanostructured surfaces may be created to increase surface area and drug-carrying capacity. (3) Absorbable carrier-based drug-eluting stents (e.g., Cre8): These employ non-polymer absorbable carriers (e.g., liposomes, amino acids) to load and release the drug. These carriers degrade rapidly after drug elution without leaving polymer residues. Compared to biodegradable polymer DES (BP-DES), PF-DESs entirely avoid potential inflammatory reactions caused by polymer degradation products. As a result, they may theoretically allow for even faster endothelialization and shorter required DAPT duration [72].

#### Critical clinical evidence: LEADERS FREE trial

The LEADERS FREE trial represents a landmark study of PF-DES in HBR patients and is the first large-scale RCT specifically designed for this population [10]. The trial enrolled 2,466 HBR patients (mean age 75 years), who were randomized to receive either a polymer-free biolimus A9-eluting stent (PF-BES, BioFreedom) or a similarly designed bare-metal stent. All patients received 1 month of DAPT, followed by single antiplatelet therapy thereafter. At 1 year, the PF-BES group showed significantly lower rates of the primary safety endpoint (cardiac death, myocardial infarction, or stent thrombosis) compared to the BMS group (9.4% vs. 12.9%; HR = 0.71; 95% CI, 0.56–0.91; P = 0.005). The primary efficacy endpoint (clinically indicated TLR) was also significantly reduced in the PF-BES group (5.1% vs. 9.8%; HR = 0.50; 95% CI, 0.37–0.69; P < 0.001). These benefits were sustained at 2-year follow-up, with both safety and efficacy endpoints remaining significantly in favor of the PF-BES. Of particular note, the rate of definite stent thrombosis was significantly lower in the PF-BES group (1.0% vs. 2.2%; P = 0.02), a finding that challenges the conventional view and suggests that, even with only 1 month of DAPT, modern PF-DES may offer superior thrombotic safety compared to BMS in HBR patients.

### BRS

#### Technical principles and characteristics

BRS represents an innovative direction in coronary intervention, designed to provide temporary mechanical support and drug delivery before being fully absorbed, thereby restoring the natural structure and function of the vessel [73]. In theory, BRS eliminate long-term risks associated with permanent implants, such as chronic inflammation, late stent thrombosis, and interference with future revascularization. The most extensively studied BRS is the Absorb everolimus-eluting scaffold made of polylactic acid. Other materials, including magnesium alloys and polyesteramides, have also been used in BRS development [74]. However, first-generation BRSs exhibit notable limitations compared to metallic DES: thicker struts (150 µm vs. 80–90 µm), which may impede hemodynamic flow; weaker and shorter-lasting radial strength (lasting approximately 6 months); stringent implantation requirements, with improper expansion increasing the risk of scaffold fracture; and a prolonged absorption period (2–3 years), which may provoke inflammatory reactions during degradation.

#### Evidence and limitations of BRS in patients with HBR

Evidence supporting the use of BRS in high-bleeding-risk patients remains extremely limited. A primary reason is that first-generation BRS (such as Absorb) required prolonged DAPT, typically for at least 12 months, which contradicts the needs of HBR patients [75].

The ABSORB III trial compared the Absorb BRS with a metallic everolimus-eluting stent (EES) in 2,008 patients. Results showed that the BRS group had significantly higher 3-year target lesion failure rates than the EES group (13.4% vs. 10.4%; P = 0.03), along with an increased rate of scaffold thrombosis (2.3% vs. 0.7%; P = 0.01) [76]. These unfavorable outcomes led to the withdrawal of the Absorb scaffold from the market and indicated that first-generation BRS are unsuitable for HBR patients. Next-generation BRS are under development to address these limitations through improved design features, including thinner struts (< 100 µm), faster absorption rates, and enhanced mechanical performance. However, these devices remain investigational at present [77]. Given the current evidence, BRS are not recommended for routine use in HBR patients outside strictly conducted clinical trials. Reflecting this, the 2018 ESC Revascularization Guidelines assign a class III recommendation (not recommended) for BRS, particularly in HBR populations [37].

### Dedicated HBR stent system

#### BioFreedom stent system

The BioFreedom stent is currently the only stent system specifically developed and approved in the European Union for high-bleeding-risk patients [15]. Its design features include: (1) a stainless steel platform (strut thickness 112 µm); (2) a polymer-free surface with microstructured texture; (3) loading of the lipophilic drug biolimus A9; and (4) rapid drug release (> 90% eluted within 28 days).

The LEADERS FREE and LEADERS FREE II trials established the favorable profile of the BioFreedom stent in HBR populations [10, 78]. Based on this evidence, BioFreedom became the first DES to receive both European and US FDA approval for use in HBR patients with DAPT as short as 1 month. In a subgroup analysis of HBR patients requiring oral anticoagulation, the LEADERS FREE sub-study demonstrated that the polymer-free biolimus-eluting stent significantly reduced the risk of major adverse events compared to BMS (HR = 0.65; 95% CI, 0.43–0.98) [30]. This finding is particularly relevant for patients with conditions such as atrial fibrillation who require long-term anticoagulation.

#### Other stent systems optimized for HBR

Synergy BP-DES features a platinum-chromium platform with ultra-thin struts (74 µm) and a biodegradable polymer that fully releases the drug and degrades within 3–4 months. The EVOLVE Short DAPT study evaluated the performance of the Synergy stent in HBR patients after 3 months of DAPT. Results indicated that discontinuing the P2Y12inhibitor after 3 months did not increase the risk of adverse events [79]. Resolute Onyx DES, although using a permanent polymer, incorporates innovative CoreWire technology that enables ultra-thin struts (81 µm) and optimized drug-release kinetics. The ONYX ONE study demonstrated its safety and efficacy in HBR patients with only 1 month of DAPT [22]. Cre8 EVO adopts a polymer-free design, with the drug embedded into micro-reservoirs on the stent surface for controlled elution. These innovative stent systems offer diverse options for HBR patients, accommodating varying clinical needs and lesion characteristics. Clinical decision-making should be tailored to individual patient profiles, lesion complexity, and the available evidence.

Beyond the above platforms, the XIENCE series EESs (XIENCE 90 and XIENCE 28) are another pivotal option tailored for HBR patients. Together with the BioFreedom stent, they are the two coronary stents approved by the FDA for short-duration DAPT strategies in patients with HBR, carrying important clinical application value.

XIENCE 90 and XIENCE 28 belong to durable polymer EESs with ultra-thin strut design. XIENCE 28 is approved for a 28-day DAPT regimen, while XIENCE 90 is indicated for a 90-day short-course DAPT scheme, forming a complete graded stratified treatment system for HBR clinical subtypes: 1-month ultra-short DAPT + XIENCE 28 for high-bleeding-risk ACS-HBR patients; 3-month short DAPT + XIENCE 90 for stable CAD-HBR patients with moderate bleeding risk. Relevant clinical trials have validated their safety and efficacy in the setting of abbreviated DAPT. In dedicated cohorts of HBR patients, both stents maintained low rates of stent thrombosis and target vessel revascularization under short-term DAPT, without a significant rise in major bleeding events. Their optimized polymer formulation and controlled everolimus release kinetics facilitate timely endothelialization of the vessel wall, which lays a structural foundation for safely shortening DAPT duration. Collectively, these optimized HBR-specific stent systems are supported by 2023 ESC DAPT guidelines with class IIa level A evidence for individualized selection in ACS and stable CAD HBR patients.

These innovative stent systems, including XIENCE series stents, offer diverse options for HBR patients, accommodating varying clinical needs and lesion characteristics. Clinical decision-making should be tailored to individual patient profiles, lesion complexity, ischemic and bleeding risk stratification, and the available evidence.

### DCB strategy for HBR patients

#### Technical advantages of DCB for HBR population

DCB angioplasty has emerged as a novel and favorable interventional strategy specifically suitable for HBR patients, fundamentally addressing the core clinical dilemma of balancing ischemic protection and bleeding complications in HBR patients. Unlike BMS and various DES platforms that require permanent vascular implantation or residual polymer scaffolds, DCB delivers lipophilic anti-proliferative drugs (typically paclitaxel) to the coronary vessel wall via transient balloon inflation, achieving uniform local drug deposition and sustained inhibition of neointimal hyperplasia without leaving any foreign implant material in the vessel lumen [80]. This unique implant-free design endows DCB with unparalleled advantages for HBR patients in terms of antiplatelet therapy management. Traditional BMS and DES require mandatory short-term or medium-term DAPT to prevent stent thrombosis, even for optimized HBR-specific stents. In contrast, DCB-only intervention eliminates the risk of stent thrombosis caused by residual struts and polymer inflammation, thereby significantly reducing the dependence on DAPT [81]. Clinically, DCB procedural guidelines and real-world consensus support an ultra-short DAPT regimen of only 2–4 weeks after successful DCB angioplasty, which is far less stringent than the 1–3 month minimum DAPT requirement for modern optimized DES and BMS. This substantial reduction in antiplatelet exposure duration effectively minimizes major bleeding risk, making DCB a highly competitive alternative strategy for HBR patients with extremely high bleeding susceptibility [41].

#### Clinical evidence comparing DCB and DES in HBR patients

Multiple prospective clinical trials and real-world cohort studies have systematically compared the safety and efficacy of DCB versus DES in HBR populations, verifying that DCB can achieve comparable or even superior PCI outcomes with abbreviated DAPT regimens. The PICCOLETO trial was a pivotal head-to-head study comparing DCB and second-generation DES in elderly HBR patients (≥ 75 years old) with *de novo* coronary lesions [82]. A total of 528 HBR patients were randomized to receive either paclitaxel-coated balloon angioplasty or everolimus-eluting DES implantation, with all patients receiving a unified 4-week ultra-short DAPT regimen. The 12-month follow-up results demonstrated no significant difference in the primary composite endpoint of cardiac death, myocardial infarction, and TLR between the DCB group and DES group (8.3% vs. 9.1%; P = 0.81). Notably, the DCB group exhibited a numerically lower rate of major bleeding events (BARC 2–5) than the DES group (4.2% vs. 7.6%; P = 0.13), attributing to the complete elimination of implant-related chronic inflammation and reduced antiplatelet therapy demand. The 2-year follow-up data further confirmed the sustained efficacy of DCB, with comparable target vessel failure rates and no late thrombotic events observed in either group [83]. A dedicated HBR subgroup analysis of the REST DCB registry, a large-scale multicenter real-world study, enrolled 1,896 HBR patients undergoing DCB-only or DES intervention [84]. Subgroup results showed that under standardized short-duration DAPT, the DCB group achieved equivalent anti-restenotic efficacy to new-generation DES, with similar rates of TLR (3.9% vs. 4.5%; P = 0.49) and definite thrombosis (0.8% vs. 1.1%; P = 0.57). More importantly, multivariate regression analysis identified DCB intervention as an independent protective factor against major bleeding complications in HBR patients (HR = 0.62; 95% CI, 0.41–0.93; P = 0.02), further validating its bleeding safety advantages. In terms of lesion adaptability, DCB shows unique superiority in small vessel lesions and diffuse in-stent restenosis, which are common in elderly and comorbid HBR patients [85]. For these high-complexity lesions, DCB avoids the risks of strut malapposition, chronic vascular irritation, and repeated stent implantation associated with DES, while maintaining satisfactory long-term vascular patency.

#### Clinical positioning and applicable scenarios of DCB for HBR patients

Based on the current high-level clinical evidence, DCB has become a reliable and optimized interventional strategy for HBR patients, complementing the existing stent systems and enriching the individualized treatment system for HBR populations [86]. Compared with mainstream DES and BMS, the core clinical value of DCB lies in its ultra-low bleeding risk while maintaining equivalent PCI efficacy, which solves the unmet clinical need of HBR patients with ultra-HBR who cannot tolerate even 1-month DAPT [87]. Consistent with accumulating real-world and trial evidence, the 2023 ESC Focused Update on DAPT and Revascularization provides a class IIb level B recommendation for DCB-only intervention combined with 2–4 week ultra-short DAPT in high bleeding-risk patients. The current clinical consensus supports prioritizing DCB intervention for specific HBR subgroups, including elderly patients (≥ 80 years), patients with severe chronic kidney disease, patients with a history of recurrent bleeding, and patients requiring long-term oral anticoagulation combined with HBR [88]. For these populations, DCB-based ultra-short DAPT strategy (2–4 weeks) can effectively balance ischemic and bleeding risks, achieving optimal clinical benefits. Therefore, clinical decision-making should follow individualized assessment: for lesions that do not require long-term mechanical vascular support, DCB is a preferred alternative to DES/BMS for HBR patients; for complex lesions requiring mechanical scaffolding, optimized short-DAPT DES systems (BioFreedom, XIENCE series) remain the first choice.

## Conclusions

This review systematically constructs a whole-process individualized precision management framework for HBR patients undergoing PCI, realizing the synthetic integration of risk stratification, procedural optimization, device selection and pharmacological de-escalation, rather than simple summary of scattered studies. The management paradigm of HBR patients has completed the transformation from traditional empirical generalized treatment to modern evidence-based precise balanced treatment.

Accurate HBR screening and quantitative risk stratification based on ARC-HBR criteria and validated scoring systems are the foundational premise of individualized treatment. Optimized perioperative procedures dominated by radial access and intravascular imaging guidance effectively reduce iatrogenic bleeding risk. Innovative interventional devices including PF-DES, BP-DES and DCB break through the limitations of traditional BMS, providing diversified device options for short-duration DAPT strategies. Stratified de-escalated antithrombotic therapy (including shortened treatment duration, downgraded drug intensity, and simplified dual-antithrombotic regimen for atrial fibrillation patients) achieves precise balance of ischemic and bleeding risks.

Critical analysis of existing evidence shows that current HBR management still has unresolved deficiencies: insufficient evidence for ultra-high-risk special subpopulations, lack of long-term follow-up data of short DAPT strategies, and limited predictive accuracy of existing risk scoring systems. Future research directions will focus on novel antithrombotic drugs (FXI inhibitors), AI-assisted precise risk prediction, and individualized strategies for special comorbid populations, to further improve the safety and efficacy of HBR patient revascularization.

In conclusion, through systematic integration of standardized risk assessment, optimized perioperative techniques, innovative interventional devices and individualized antithrombotic regimens, clinicians can achieve safe and effective revascularization for complex HBR patients, effectively reducing bleeding complications while ensuring ischemic protection, and ultimately improving long-term clinical prognosis and quality of life of this high-risk population.

## Data Availability

The data used to support the findings of this study are available from the corresponding author upon request.

## Abbreviations

PCI: percutaneous coronary intervention
HBR: high bleeding risk
DAPT: dual antiplatelet therapy
CAD: coronary artery disease
IVUS: intravascular ultrasound
OCT: optical coherence tomography
